# Development of a workplace breastfeeding support practice model in South Africa

**DOI:** 10.1186/s13006-024-00638-9

**Published:** 2024-05-06

**Authors:** Lynette Carmen Daniels, Xikombiso Gertrude Mbhenyane, Lisanne Monica Du Plessis

**Affiliations:** Division of Human Nutrition, Department of Global Health, Faculty of Medicine and Health Sciences, Stellenbosch University, Cape Town, South Africa

**Keywords:** Breastfeeding, Workplace, Practice model, Support

## Abstract

**Background:**

Globally, mothers have identified work as one of the main obstacles to exclusive and continued breastfeeding. The support a woman receives in her workplace in terms of workplace arrangements can be critical to enable women to continue breastfeeding. This study aimed to develop and assess the face validity of a practice model to support exclusive and continued breastfeeding in workplaces in the Western Cape, South Africa.

**Methods:**

An explanatory, sequential, mixed-method research design, was conducted (June 2017 to March 2019) in three distinct phases. Phase one employed a quantitative, descriptive, cross-sectional study design. Phase 2 used a qualitative, multiple case study. Phase three involved the development and face validity of a practice model to support exclusive breastfeeding in workplaces. The face validity included two Delphi rounds for experts to provide input on the draft practice model. This paper will only report on phase 3 of the study. The practice model was developed, drawing on the analysis of data from phases one and two and using programme theory approaches and a logic model.

**Results:**

The practice model was positively perceived. Participants viewed it as informative, well designed and easy to follow, even for those not knowledgeable about the subject. It was viewed as an ideal tool, if accompanied by some training. Participants were positive that the model would be feasible and most commended the tiered approach to implementation. They felt that workplaces would be more open to a step-by-step approach to implementation and if only a few activities are implemented it would be a start to make the work environment more conducive for breastfeeding employees. There were mixed opinions regarding commitment; a few participants mentioned commitment as a challenge they anticipated in the male-dominant environments in which they worked. The provision of space for breastfeeding at the workplace was also highlighted as a potential challenge.

**Conclusions:**

Advocacy around creating an enabling workplace environment for breastfeeding is needed. The practice model has the potential to be internationally relevant, locally applied and may be of particular use to workplaces that want to initiate and/or strengthen breastfeeding support.

## Background

Returning to work is often considered an obstacle to exclusive and continued breastfeeding. It is a major reason for mothers not breastfeeding, or for ceasing to breastfeed early. Several factors may influence the duration of breastfeeding once the mother returns to full time employment. For instance, workplace support in terms of providing breastfeeding time and space, support at home and in the community, the attitudes of employers and colleagues towards breastfeeding employees and employment conditions and workplace arrangements. For many mothers, the lack of workplace support for breastfeeding makes working incompatible with breastfeeding [1].

The creation of an enabling workplace environment for breastfeeding may assist mothers to continue breastfeeding. Enabling interventions operate to remove structural and societal barriers that interfere with a mother’s ability to breastfeed optimally [2]. Globally, workplace support for breastfeeding is progressively seen as a cost-effective investment to increase employee morale, minimize absenteeism and reduce staff turnover [2, 3]. Workplace interventions like providing lactation rooms and breastfeeding breaks are low-cost strategies [2] that can improve the duration and continuation of breastfeeding globally and in South Africa.

South Africa is a upper middle income country with low exclusive breastfeeding (EBF) rates. In South Africa only 32% [4] of infants under the age six months are exclusively breastfed, contrary to the recommendation that children under six months be exclusively breastfed. The percentage of children exclusively breastfed also decreases with age from 44% of infants age 0–1 month to 24% of infants age 4–5 months. Twenty five percent of infants under six months are not breastfed at all [4]. Mixed feeding before six months is a well-documented social norm in South Africa, closely related to beliefs that breastmilk is insufficient to nourish a child [5]. Multi-level barriers to EBF exist in South Africa. At an individual level milk insufficiency beliefs and incompatibility of EBF with schooling and employment. At a community level, norms around mix feeding including traditional and cultural beliefs that exist within communities that inform mothers infant feeding decisions [5]. Mothers face pressure to adhere to family traditions and decisions relating to infant feeding [5]. Also, with the high level of fathers being absent in many households, buying commercial milk formula is one way that families pressure males to take responsibility [6]. With this backdrop and the growing number of women in their childbearing years taking up employment, it is essential that support for breastfeeding in the workplace is reinforced and that institutional workplace challenges are addressed.

Workplace breastfeeding support and promotion models, interventions and case studies have been reported in other contexts like Thailand, Indonesia, United States, Bangladesh and Kenya [7–10]. In South Africa the South African National Department of Health (NDOH) developed a guidance booklet supporting breastfeeding in the workplace a guide for employers and employees [11]. However, to date, in South Africa there is no practice model to guide employers in supporting breastfeeding in the workplace with a set of comprehensive programme activities to implement, links and access to national and international resources and visually depicting the underlying theory of the programme. Owing to the gap in the literature the objective of this study was to develop and test the face validity of a practice model to support EBF in designated workplaces (employers who employ 50 or more employees) [12] in the Breede Valley sub-district, Western Cape Province in South Africa.

Providing workplaces with a validated tool to create an enabling workplace environment for the practice of breastfeeding, may ensure that infants are provided with exclusive and prolonged breastfeeding. This, in turn, may contribute to infants receiving the highest, most attainable standard of feeding with numerous health benefits.

## Methods

To inform the development of the practice model, an explanatory, sequential, mixed-method research study was adopted. The research was conducted in Worcester, Breede Valley sub-district in the Western Cape Province, South Africa. The setting was selected based on designated workplaces being present in Worcester area that represent linkages with various levels, namely local, regional as well as national (retail stores and large commercial food companies).

**Fig. 1:**
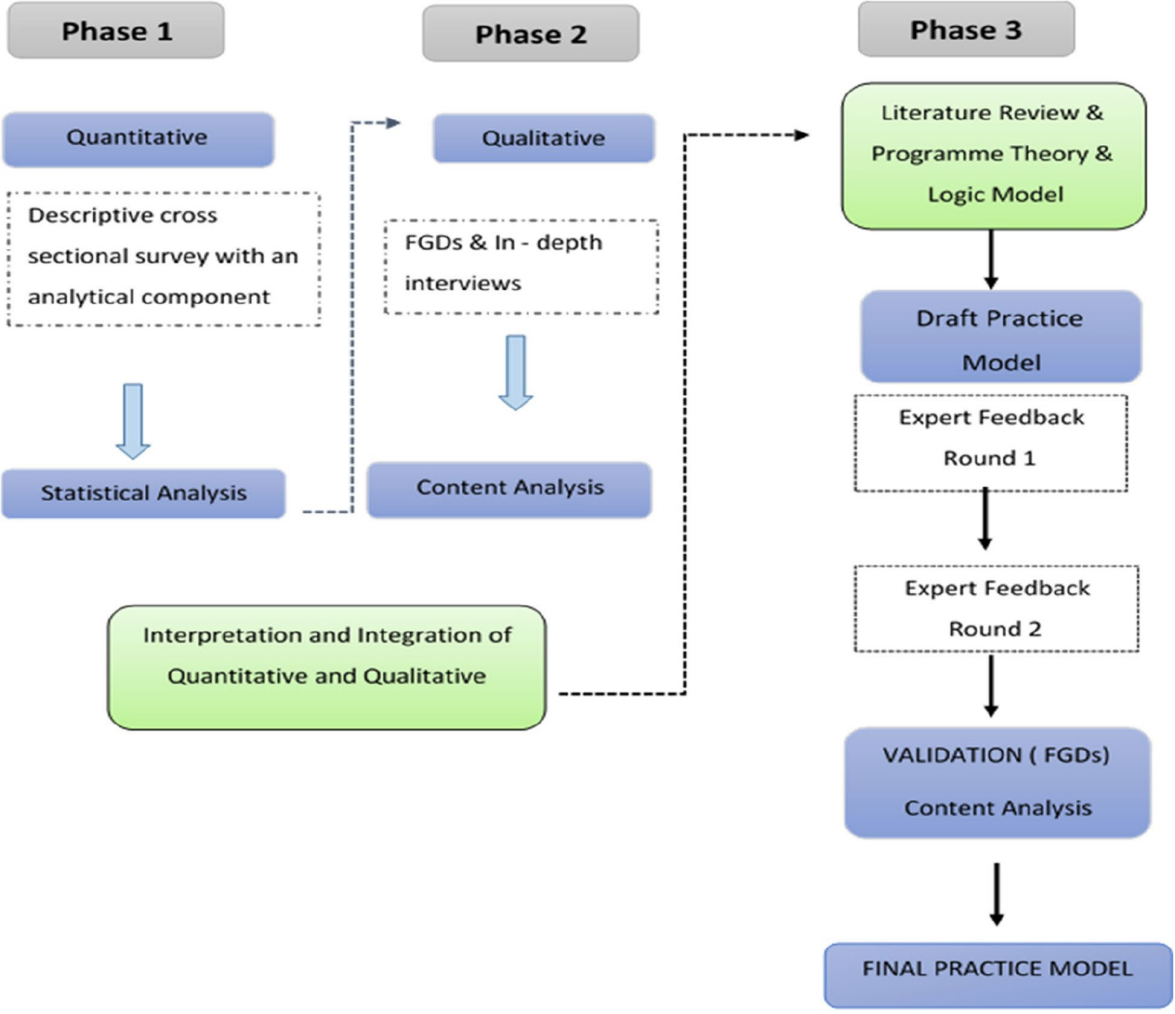
Phases of the research study

The research was conducted in three distinct phases (Fig. 1). Phase one employed a quantitative, descriptive cross-sectional study design, using an online survey to assess current breastfeeding support practices and has previously been reported [13]. Phase 2 was a qualitative, multiple case study. Data was collected at nine purposively selected workplaces from the manufacturer, retail and public sector, using focus group discussions (FGDs) with employees and in-depth interviews with managers. FGDs were also conducted with employed breastfeeding mothers from designated workplaces who exclusively or predominantly breastfed their children for any period up to six months. This paper will elaborate only on phase three of the study, the model development and face validation phase. The research received ethical approval (S17/04//089) from the Stellenbosch University Health Research Ethics Committee.

### Review of the evidence regarding workplace breastfeeding interventions and breastfeeding outcomes

A critical review of the literature in the field of workplace breastfeeding support interventions and breastfeeding outcomes were conducted. A search was conducted on PubMed using the following key words: (breastfeeding OR breast feeding OR lactation) AND (work OR workplace OR employment) AND (intervention OR support) AND (duration OR continuation OR exclusive OR rates). The literature consulted included published articles from 2008 through 2019 listed in PubMed. The PubMed search article titles was assessed. Sixteen selected article abstracts, and full text were further screened of which seven were excluded (two was qualitative studies, three was systematic reviews and two were excluded as the main outcomes measured were not breastfeeding duration, continuation and exclusivity). This yielded a total of nine articles included. Amongst the material a review article [14] on worksite lactation accommodation was found. The review article was reviewed, and five similar articles as yielded by the selected PubMed search article was found. From the critical review of the literature, it was concluded that workplace breastfeeding interventions and support services are responsive to breastfeeding outcomes and practices in terms of increased rates of breastfeeding duration, continuation and exclusivity [7, 14–20]. There is a lack of randomised control trials relating to breastfeeding support in the workplace as most studies extracted reflected cross-sectional surveys. Not all studies consistently found significant associations with all breastfeeding interventions and breastfeeding outcomes assessed. This can be attributed to possible confounding variables that may have been present e.g. lack of family support, cultural and maternal belief of the mother, the mother`s low self – efficacy for breastfeeding.

### Identifying main elements from phase one and two for inclusion in the practice model

Phase one and phase two were analysed, interpreted and the main issues arising were identified for inclusion in the model. Phase one analysis involved statistical analysis of the quantitative data. Phase one findings revealed that provision of a private space for breastmilk expression was uncommon [13]. Allowing time for breastfeeding and promoting breastfeeding amongst the employees were also not commonly practiced [13]. The provision of breastfeeding time in South Africa is recommended according to the Basic Conditions of Employment Act, Code of Good Practice on the Protection of Employees during Pregnancy and After the Birth of a Child Sect. 5.13 [21]. Needs identified by managers related to physical space, a regulatory framework, communication, education and information. From phase one of the study, the following pertinent issues raised were taken up in the practice model: provision of breastfeeding time and private space, education and communication.

Phase two analysis of the FGDs with employees and in-depth interviews with the managers involved content analysis using the Atlas ti programme. Phase two findings revealed the absence of private space and time for expressing breastmilk as major challenges for women returning to work. The lack of communication between employers and employees regarding their needs, policies regulating their return from maternity leave, as well as unsupportive attitudes on the part of staff and co-workers were highlighted. The employed mothers who successfully combined breastfeeding and work had a strong belief in the benefits of breastfeeding and this motivated them to continue breastfeeding with work. Therefore, the provision and promotion of breastfeeding time and space as well as communication (conducting a return-to-work consultation after maternity leave) were included in the practice model. Also included in the model were addressing unsupportive attitudes and increasing belief in breastfeeding among employees by enhancing knowledge of the benefits of breastfeeding and the recommended breastfeeding time through education. Conducting a needs assessment amongst women to assist managers with planning and coordination was also taken up in the practice model.

### Integrated programme theory and program logic models to draft the practice model

A programme theory describes how and why a programme is supposed to work [22]. The process of developing a programme theory promotes evidence-based thinking and provides a clear understanding of how change will occur. It also describes the beliefs and assumptions that underlie the choice of activities, thereby making the results more credible [22]. By reviewing the literature, the underlying linkages and secure evidence of the mechanism of change that would lead to improved breastfeeding duration and EBF rates amongst employees could be explained. The process involved moving continually between theory and practice to develop the practice model programme theory. A logic model is a commonly used tool for illustrating an underlying programme theory. The logic model was selected for the mentioned reason and also as the logic model would provide stakeholders with an easy-to-follow clear visual representation and clear specific guidance in terms of what needs to be provided (input and responsibilities) and the potential gains it holds. The following components are included in a programme logic model 1) Inputs: any resource or material used by the programme to enable its activities e.g. private hygienic space with resources 2) Activities: any services or treatments provided by the programme e.g. engage in interpersonal communication a return to work consultation 3) Outputs: amount of activity provided, described in quantifiable terms e.g. number of employees aware of breastfeeding space 4) Outcomes: any characteristics of the participants that, according to the programme theory, are expected to change as a result of the participants’ receiving services e.g. improved staff wellness and better work life integration practice 5) Impact: the ultimate intended change e.g. cost saving in terms of retaining staff [23–25].

### Delphi rounds

A list of experts in the fields of infant feeding, breastfeeding, human resources management (private and public sectors), academia, child health and behavioural and organizational development was developed. The experts met the requirements of having knowledge and experience of breastfeeding, infant feeding, or human resource management in an organization. An email was sent to the experts to invite them to take part in the study as an expert panel member. Sixteen experts were invited and 11 (69%) participated in the two modified Delphi rounds. The Delphi process and rounds allowed the researcher to gather the opinions of a panel of experts without having to bring them together physically, thereby saving time, cost and effort. The process is free from social pressure and individual dominance and conducive to independent thinking and the gradual formulation of judgement. Of the 11 expert members, ten were female and one male. Four of the expert members were from the academic/research sector and three were International Board-Certified Lactation Consultants. The remaining expert members comprised a provincial health department official, a UNICEF nutrition specialist, one human resource practitioner from the private sector and one from a provincial organizational development department, industrial psychologist.

A graphic designer assisted with the drafting and refining of the practice model. The first version of the practice model was supported by an additional one-page information sheet providing guidance as to the inputs and activities that the employer must provide and perform. The Delphi round one questionnaire consisted of open-ended questions relating to the elements in the developed practice model: inputs, activities, outputs, outcomes, linkages/connections between inputs, activities, outputs and outcomes, strengths, weaknesses, achievability/realistic to implement, challenges, design, use of wording and any recommendations and improvements. Content analysis techniques was used to the open-ended input by grouping similar items together and summarizing the comments received. These were discussed by the research team. Thereafter the practice model was amended. Under the inputs, the identification of a breastfeeding champion, including trade unions, lactation consultants and breastfeeding counsellors as well as other international toolkits was added. Two activities were added to the model: one was maternity leave provision, as this was believed to contribute to the achievement of the stated long-term outcome of increased breastfeeding duration. The provision of antenatal education by the employer was removed and changed to the inclusion of time for pregnant employees to participate in antenatal visits/classes/clinics, which was added under the education heading. Granting pregnant women time off to participate in antenatal preparation is included in Sect. 5.12 of the Code of Good Practice on the protection of employees during pregnancy and after the birth of a child [21]. Listed activities linked to legislation was highlighted with the text “legal obligation /recommendation to provide”. Also, additional long-term outcomes were added, and more images included under impact.

**Fig. 2:**
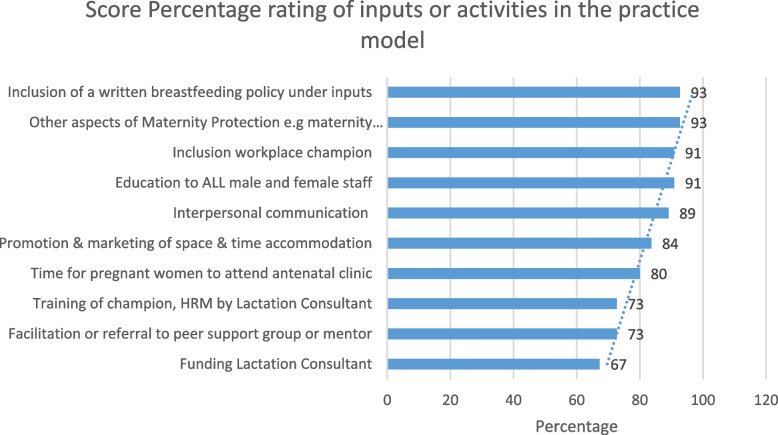
Score percentage rating for the inputs and activities in the practice model

The improved, second version of the model was sent to the experts. A set of questions mostly using scoring/ranking techniques was used to gain consensus on the amended practice model inputs, activities and outcomes and the connections between them. The round two questionnaire consisted of ten questions relating to the importance of the input and activities used in the model on a one-to-five rating scale of 1 = least importance and 5 = high importance. Also, ten four-point Likert-scale statements and four four-point rated (poor, fair, good, excellent) questions relating to the overall design. To determine the importance of the inputs, a mean score and mean percentage score for each stated input were calculated (Fig. 2). Inputs and activities were judged less valid if there was less consensus on their importance. For the input and activity variables, a mean score of 70% (3.5 out of 5) was deemed to be valid for inclusion in the practice model. Therefore, funding for a lactation consultant was removed and a written breastfeeding policy statement as well as the provision of maternity leave benefits were both added to the model, as both scored an overall percentage of 93% (4.65/5).

After the input from the Delphi round two, the final amendments to the model were made. Following on, the practice model face validity was tested by presenting it to the to the nine workplaces that participated in phase two, during four focus group discussions and one in-depth interview. These participants (*n* = 16) included human resource practitioners (*n* = 5), occupational health nurses (*n* = 2), social workers (*n* = 2) managers (*n* = 6) and personal assistant (*n* = 1). The majority of the participants was from the public sector (*n* = 8), manufacturing sector (*n* = 5) and retail sector (*n* = 3). The Atlas ti programme was used to analyse the qualitative data. The analysis was mainly deductive (having a pre-prepared structure), but also partly inductive, in terms of which emerging themes were built and developed.

## Results

**Fig. 3:**
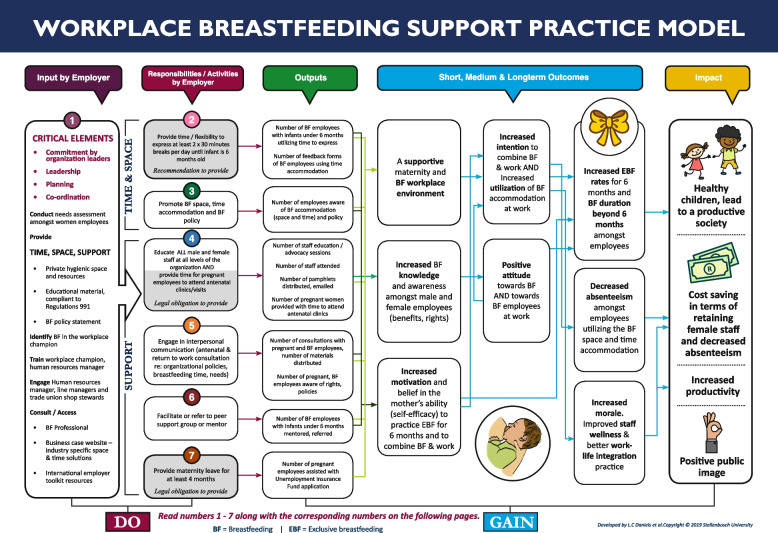
Workplace breastfeeding support practice model

The practice model (Fig. 3) projects a simple logic model flow, making clear connections within the proposed theory of change. The programme theory is based on the Theory of Planned Behaviour [25] which states that three categories of belief guide human action-oriented behaviours: the outcomes of performing the behaviour (behavioural beliefs – what you feel, think, and the importance of the behaviour = attitude), the expectations of significant others (peers, supervisors) in relation to the behaviour (referent beliefs, how others view the behaviour = subjective norm) and the presence of factors that facilitate or hinder the behaviour (control beliefs = perceived behavioural control).

The programme theory in the practice model relies on working mothers’ attitude towards breastfeeding and the uptake of available breastfeeding accommodation (space and time) at the workplace. The uptake of available breastfeeding accommodation depends on a working mother’s intention to combine breastfeeding and work. Intention is influenced by the working mother’s belief in her ability to successfully combine breastfeeding and work (self-efficacy) and her motivation to do so (control belief). It is also influenced by the working mother’s attitude towards breastfeeding and, more specifically, breastfeeding for an extended period (behavioural belief), as well as by the support she receives from workplace supervisors, peers and co-workers (referent belief) [25].

The theory of change hinges on the following change mechanism as depicted in Fig. 3 short term outcomes, leading to the medium term and long-term outcomes: when workplace breastfeeding support is provided in terms of space, time and support (i.e. education, peer support, communications and policies), employees’ breastfeeding knowledge will increase and foster positive attitudes towards breastfeeding and breastfeeding employees. It will also increase self-efficacy and the motivation to combine breastfeeding and work, which strengthens the intention to combine breastfeeding and work, leading to increased utilization of the available breastfeeding accommodation (space). This may ultimately lead to increased breastfeeding duration and EBF rates among employees. The literature indicates the connection between the implementation of a workplace breastfeeding support programme and significant higher breastfeeding rates after implementation [7] as well as the positive correlation between the duration of breastfeeding with the level of workplace support, sufficient time and availability of appropriate space at work to express breastmilk [18]. Organizational support (Odds Ratio = 1.80) increases the odds of EBF by nearly twofold [20].

### Input by employer

The practice model starts off by identifying the critical elements required from the employers. Commitment by organizational leaders, leadership, planning and co-ordination were identified as key elements for the model to succeed.

### Responsibilities/activities by employer

The model flows from left to right, starting with the model inputs and responsibilities/ activities that should emanate from the employer. Among the activities and inputs included in the practice model, the elements of time, space and support are paramount. These three elements have been regarded in the literature as the critical elements of a breastfeeding friendly workplace since 1993 [26]. The activities included in the practice model can be viewed in the below Table 1. These activities scored more than 70% by the experts in the second Delphi round and meant consensus for inclusion in the practice model.

**Table 1:** Activities included in the final practice model

Providing the recommended breastfeeding time and flexibility (allow 2 × 30 min breaks per day until infant is 6 months old)
Promotion of breastfeeding space, time accommodation and breastfeeding policy
Education of all male and female staff at all levels of the organization and providing pregnant women time to attend antenatal clinic visits
Interpersonal communication (antenatal and return-to-work consultation) regarding organizational policies, breastfeeding time, and the mother’s needs
Facilitation or referral to peer support group or mentor
Provision of the legislated four months maternity leave

### Outcomes

The model also expands on the expected outcomes, which were based on the literature review and the programme theory of change. The six listed activities included in the practice model are intended to lead to short-term, early changes (i.e. creation of a supportive breastfeeding environment, increased breastfeeding knowledge, increased motivation and self-efficacy to practice EBF and combine breastfeeding and work), which in turn set in motion changes in the medium term (i.e. increased intention to combine breastfeeding and work, increased utilization of breastfeeding accommodation, positive attitudes towards breastfeeding and breastfeeding employees). This is expected to result in the long-term outcomes of increasing breastfeeding duration and exclusivity rates for the first six months of babies’ lives among employees.

### Outputs

Outputs quantify the services provided and describe what the specific activities will produce. The set indicators for each activity were developed by the researcher with inputs form the expert panel members during the two Delphi rounds. The outputs for each activity are portrayed in Fig. 3, which guides the monitoring aspects of the model. A potential challenge with measuring the outputs and the outcome may include the additional administrative load in monitoring the various aspects.

### Impact

Impact is the fundamental intended or unintended change occurring in organizations, communities or systems as a result of programme activities within seven to ten years [23]. The impacts in terms of societal gains (healthy children lead to a productive society) and workplace gains (cost saving in terms of retaining female staff and decrease absenteeism, positive public image) are made explicit in the model.

The model is accompanied by a three-page information sheet providing further explanations on the inputs and activities /responsibilities that needs to be provided by the employer and links to various resources that can assist with the support process.

### Qualitative results of the face validity of the practice model

To determine the face validity of the practice model after the two Delphi rounds, four FGDs and one in-depth interview were conducted. Participants (*n* = 16) had sufficient opportunity to review the practice model, as the protected document was emailed a few days prior to the discussions to enable participants to engage with the content. The Human Resource practitioners, managers` and occupational health nurses of the nine participating workplaces were invited to attend a focus group discussion in February or March of 2019. Gaining the input of the stakeholders was deemed important in order to gauge the end user’s perception of the model. The input of stakeholders in the development and the face validity of the model serves to improve the quality and also encourage the use of it [24].

Three main themes were established namely, perceptions of developed practice model, challenges to implementation of the practice model and suggested changes to the model.

The practice model was positively perceived by the majority of the participants. The participants viewed it as informative, well designed and easy to follow ‘*the layout is good’, ‘It`s clearly set out and understandable`*. The model was viewed as an ideal tool, if accompanied by some training of how to apply and use it. Participants were positive that implementation of the model was achievable. The model was regarded as informative and insightful, even for someone who are not knowledgeable about the topic. Participants felt the model provided a basic guideline of how to implement breastfeeding support in the workplace.

‘It’s an incredible model … but we know that we must make place for breastfeeding, we know that there are certain laws, but we don’t necessary do it and we don’t know how. So this is actually the how ….` HR manager, manufacturer, male

The tiered approach (selecting a few activities to pilot first) was perceived as the best way to approach implementation. Participants felt that workplaces would be more open to a step-by-step approach.

‘… for management to get into the idea… if it’s in steps then I think they will be more open to the idea.` HR manager, manufacturer, female

In terms of the three-page information sheet some participants felt at first glance that there was a lot of information to process. Others considered the information sufficient. Most participants appreciated the degree of detail in the information sheet and described it as informative, clearly set out and reader friendly.

‘So I think that it’s good that the explanations is there. Because we are being told: “consult people”, but then you are, where should I begin, who must I look for, who else must I approach?` HR manager, retail, female

Commitment on the part of organization leaders, an important element mentioned in the model, was viewed as critical for the model to succeed. There were mixed opinions regarding commitment; a few participants mentioned commitment as a challenge they anticipated in the male-dominant environments in which they worked.

‘You need the commitment from the leaders or the management team to buy into this model, to have things in the workplace like that. So, I think that is aimed …. Most of them at our company are men so they don’t usually understand the importance of breastfeeding and things like that. So, I think that to me will be a challenge.’ HR manager, manufacturer, female

Others stated that their workplace would commit to something like that, as it stands to benefit both employees and the employer. It is therefore essential that organizational commitment be developed for successful breastfeeding support. Other challenges that workplaces may experience with implementation of the model was voiced as the provision of space within their work environments.

## Discussion

This study aimed to develop and assess the face validity of a practice model to support EBF in designated workplaces at district level. A similar Australian-based study also developed a good practice model to evaluate the effectiveness of comprehensive primary health care in local communities [27]. This research employed a similar methodology of combining programme logic and theory-based approaches, drawing on the literature and conducting interviews to draft the practice model. Another study set out to develop a generic community health worker (CHW) logic model, proposed a theoretical causal pathway to improved performance [28]. The researchers described their model as a practical tool that offers guidance for continuous learning about what works. The value of the logic model for CHWs was that it can aid planning, draw attention to certain elements of design that are sometimes overlooked, contribute to consensus building to facilitate communication and a shared understanding of what is needed, and it can be used as a guide for improving programme implementation [28]. The reported value of the logic model for CHWs can similarly apply to the value of the practice model developed in this study, which is also based on a programme logic model.

The six activities included in the practice were providing the recommended breastfeeding time and flexibility (allow 2 × 30 min breaks per day until infant is 6 months old), promotion of breastfeeding space, time accommodation and breastfeeding policy, education of all male and female staff at all levels of the organization and providing pregnant women time to attend antenatal clinic visits, interpersonal communication (antenatal and return-to-work consultation) regarding organizational policies, breastfeeding time, and the mother’s needs, facilitation or referral to peer support group or mentor and provision of the legislated four months maternity leave. The implementation of these selected activities and workplace interventions and the impact on breastfeeding outcomes are well documented. Research reveals that women with access to breaks and space are 2.3 times more likely to EBF at 6 months [29] and women with an independent breastfeeding room in the workplace are less likely to discontinue breastfeeding [30]. Qualitative research found interpersonal communication as important to enhance workplace breastfeeding support [31]. A review article highlights the positive relationship between providing maternity leave and breastfeeding duration [32]. Furthermore, the literature reveals that a workplace environment that included supervisor and peer support, quiet space other than a bathroom and the frequency of breastfeeding in the workplace showed significant positive correlations (*r* = 0.26, *p* = 0.01) with a longer duration of EBF [12].

Also, the literature shows that a combination of different interventions physical resources (e.g. space) organizational resources (e.g. flexible breaks) education resources and workplace support (e.g. by establishing policy and encourage support from managers and co-workers) leads to more comprehensive breastfeeding support programmes [33]. However, the comprehensive approach is still uncommon [34]. The practice model suggests a comprehensive set of activities to be implemented.

To achieve equitable work environments, interventions should focus at the three ecological layers namely individual, interpersonal, and organizational [34]. The workplace is an organizational level in which institutional support for breastfeeding mothers can be fostered. This support interacts with individual level factors linked to breastfeeding intentions and self-efficacy. Organizational support in terms of promoting lactation rooms and flexible time to express breastmilk, is associated with longer breastfeeding duration [34]. Interpersonal factors are also important and includes support from co-workers. An effective way to promote organizational support is imparting knowledge among coworkers [34]. When evaluating the activities in the practice model it addresses these three ecological layers through education of all staff, institutional support in terms of space, time and policies which links to individual factors of intention and self-efficacy.

The practice model is distinctive as it showcases the activities and their potential pathways of change. A systematic review highlighted that interventions and their impact pathways need to be better understood and documented [34]. The systematic review suggested further studies on the adaption of workplace interventions for firms of different types and size. The practice model addresses this, as it was mainly developed for workplaces with more than 50 employees from the manufacturing, retail and public sectors.

A participatory action research study was conducted in Thailand to develop a workplace breastfeeding support model for employed lactating mothers [7]. This study compared breastfeeding rates before and after implementation of the breastfeeding support campaign. The Thailand model was also based on the three core elements of time, space and support. The process undertaken to develop the Thailand model included the creation of a breastfeeding support committee, breastfeeding support activities and educational materials for breastfeeding support campaigns in the workplace. Similarly, this practice model incorporates engagement with trade unions, human resources managers, line managers and consulting breastfeeding professionals. The implementation of the Thailand model consisted of breastfeeding education and support by nurses, midwives and/or lactation consultants as part of the breastfeeding support campaign at the workplace. Breastfeeding education and support from health professionals started from the third trimester of pregnancy and continued during hospitalisation, after giving birth, during the four- to six-week post-partum follow-up visit, one-two weeks before resuming employment, and continued after the mother’s resuming employment through at least six months. Similarly in the current study, the aspect of breastfeeding education for all levels of male and female staff was included as an activity in the practice model. The practice model additionally included facilitation and/or referral to peer support groups as part of the support. The Thailand model proved to be effective, as the breastfeeding rates after implementation were significantly higher for both EBF and any breastfeeding at six months, at levels of 0.004 and 0.033 respectively. The employed lactating mothers had a positive psychological experience from combining breastfeeding with work, and members of the committee reported positive attitudes towards breastfeeding and themselves felt good about supporting lactating workers [7].

A study by Basrowi et al. (2018) in Indonesia similarly to the current study, used the aspect of expert consensus on developing a workplace-based lactation promotion model. A three-round online survey using Delphi approach was conducted. The seven dimensions, i.e. policy and regulation, facility, education material, target participants, promotion approach, human resources, and time was highlighted as the most important actions to promote lactation in the workplace. In the final round, “maternity leave of 3–6 months” and “employees have the right to breast-pumping every 3 h” ranked as the two most important indicators regarding policy and regulation. Having a dedicated lactation room was the highest ranked indicator regarding facility dimension. Regarding education materials, benefits of breastmilk for babies ranked as the highest indicator. The top management in the company and lactation counsellor are the two highest-ranked indicators in human resources dimension. For the delivering methods dimensions, social media and interactive counselling were the two highest ranked indicators [8]. The dimensions highlighted in this study has several links with the developed practice model. The links relates to importance of breastfeeding time, providing the maternity leave period, space/facility, involvement of education, using interactive communication and the importance of top management and lactation counsellors.

A study by Garvin et al. (2013) in Southeastern Virginia aimed to assist workplaces in developing lactation support using the business case for breastfeeding resource toolkit [9]. This one-year project educated 20 businesses about breastfeeding support in the workplace and engaged 10 businesses to implement the business case for breastfeeding. The aim was to assess sustainability via documented policy and environmental changes as well as integration of the lactation support programme into the businesses infrastructure. The results indicated out of 17 businesses that engaged with the project 14 significantly increased their stage of change, development of a lactation support programme, written policies and physical and social environment changes (*p* < 0.001). A brief follow-up also indicated that all 14 businesses sustained the programme eight months after the program ended with increased stages of change, policy enforcement and physical environment (*p* < 0.05). This resource toolkit provided an effective approach in assisting and maintaining lactation support programmes in workplaces across several cities in Virginia. Similar to the Garvin et al. study there is also potential to conduct similar research in the South African context. By providing workplaces with education and training relating to the developed practice model and assessing the progression of the stages of change, physical and social environment with the aim to determine if the approach would be effective in establishing support in the workplace.

The United Nations Childrens Fund (UNICEF) Bangladesh and Kenya workplace breastfeeding case study, similar to the practice model activities of the current study, included interpersonal communication, pre-maternity leave and a back-to-work preparatory consultation. Workplace dialogue was included to sensitize employees and promote available breastfeeding breaks and space accommodation. The lessons learned from the Bangladesh case study were about the importance of involvement from leadership as well as commitment from the business to initiate the programme [10]. The practice model starts off by identifying the critical elements, with leadership and commitment considered important elements for the practice model to succeed.

A report by World Health Organization and UNICEF relating to breastfeeding family-friendly policies sets out a call for action: advocacy within business is needed and workplace policies must be adapted and strengthened. The report mentions that technical assistance is needed to build workplace capacities to implement such policies [35]. The practice model addresses these aspects through providing workplaces with practical guidance in how to build workplace capacities to support breastfeeding.

A recommendation is for a campaign for the endorsement of the practice model by all government departments in the Western Cape. The South African NDOH should consider recognition for workplaces that support breastfeeding. Government could for example provide tax breaks as an incentive for employers to create breastfeeding facilities. Furthermore, the NDOH should ensure the distribution, awareness and marketing of the breastfeeding in the workplace toolkit and guide for employers and employees. Much more advocacy around this aspect is needed.

In terms of the results and outcomes of this research, using a sequential mixed-method approach proved to be helpful in bridging the gap between research knowledge and the creation of an action-orientated model to help create a supportive workplace environment for breastfeeding. Limitations in terms of the model development process relates to the extensive logistical arrangements to set up a suitable date and time for the FGDs for the participants with high-ranking positions. The Delphi experts also held high ranking positions and were employed in occupations that are very demanding. The two Delphi rounds took three months of periodic interaction to complete. Participant fatigue was sensed, and no further rounds were therefore conducted. Another limitation can be that the research did not engage organizational change theory to develop the model in conjunction with the programme theory used. A challenge of the model may be that it may be too ambitious for some workplaces to implement. Other potential barriers to implementing the model may be the commitment from leaders may be lacking due to breastfeeding not being a priority in the organization, the male dominant work environments that do not prioritize female needs and breastfeeding. Also not able to identify a breastfeeding champion to drive the process in the workplace and providing the space for breastfeeding and the associated infrastructure. To overcome these potential challenges advocacy in workplaces around the importance and benefits of workplace breastfeeding support is needed.

## Conclusion

The practice model was positively perceived. Participants viewed it as informative, well designed and easy to follow, even for those not knowledgeable about the subject. It was viewed as an ideal tool, if accompanied by some training. Participants were positive that the model would be feasible and commended the tiered step by step approach to implementation. Challenges related to commitment from management, especially in the male dominant environments as well as the provision of a breastfeeding space. There is a need for greater advocacy around creating an enabling workplace environment for breastfeeding. If employers implement concerted activities according to the practice model, it may go a long way to assist employed mothers with EBF and longer breastfeeding duration. It additionally holds the potential outcomes for the employee in terms of increased morale, improved staff wellness and better work life integration, which benefits the workplace in terms of cost saving relating to retaining skilled female staff, decreased absenteeism and increased productivity. The practice model has the potential to be applicable nationally and relevant internationally. The model can be locally applied and may be of particular use to workplaces that want to initiate and/or strengthen breastfeeding support. Future research should explore the piloting of the practice model within the three workplace categories (i.e. retail, manufacturing and public) and exploring the perceptions of employees and employers before and after the implementation of the intervention and to evaluate the effect on breastfeeding outcomes. Research on the impact of the recommended breastfeeding time on organizational workplace practices and policies is also needed. Exploring the experiences of breastfeeding mothers employed in the informal sector is also recommended, as these mothers presumably face similar but unique challenges.

## Data Availability

No datasets were generated or analysed during the current study.

## Abbreviations

EBF: Exclusive breastfeeding
FGDs: Focus group discussions
NDOH: National Department of Health
UNICEF: United Nations Children Fund
